# M6A “Writer” Gene *METTL14*: A Favorable Prognostic Biomarker and Correlated With Immune Infiltrates in Rectal Cancer

**DOI:** 10.3389/fonc.2021.615296

**Published:** 2021-06-17

**Authors:** Changjing Cai, Jie Long, Qiaoqiao Huang, Ying Han, Yinghui Peng, Cao Guo, Shanshan Liu, Yihong Chen, Edward Shen, Kexin Long, Xinwen Wang, Jian Yu, Hong Shen, Shan Zeng

**Affiliations:** ^1^ Department of Oncology, Xiangya Hospital, Central South University, Changsha, China; ^2^ National Clinical Research Center for Geriatric Disorders, Xiangya Hospital, Central South University, Changsha, China; ^3^ Key Laboratory for Molecular Radiation Oncology of Hunan Province, Xiangya Hospital, Central South University, Changsha, China; ^4^ Department of Pathology, University of Pittsburgh School of Medicine, Pittsburgh, PA, United States; ^5^ Department of Life Science, McMaster University, Hamilton, ON, Canada

**Keywords:** m6A, *METTL14*, immune infiltration, immunotherapy, rectal cancer

## Abstract

Rectal cancer (RC) is the leading cause of tumor-related death among both men and women. The efficacy of immunotherapy for rectal cancer is closely related to the immune infiltration level. The N6-methyladenosine (m6A) modification may play a pivotal role in tumor-immune interactions. However, the roles of m6A-related genes in tumor-immune interactions of rectal cancer remain largely unknown. After an evaluation on the expression levels of m6A-related genes and their correlations with the prognosis of rectal cancer patients, we found that *METTL14* was the only gene to be significantly correlated with prognosis in rectal cancer patients. Therefore, we further observed the impact of *METTL14* expression and m6A modification on the immune infiltration in rectal cancer. Our study indicates that low expression of the m6A “writer” gene *METTL14* in rectal cancer may lead to the downregulation of m6A RNA modification, thus reducing the level of immune cell infiltration and resulting in poor prognosis. *METTL14* expression level is an independent prognostic factor in rectal cancer and is positively correlated with the immune infiltration level. Our study identified *METTL14* as a potential target for enhancing immunotherapy efficacy in rectal cancer.

## Introduction

Rectal cancer (RC) is the leading cause of tumor-related death among both men and women (1). Thus, clinicians are calling for more effective treatments for rectal cancer. Immune checkpoint inhibitors have achieved successful responses in few rectal cancer patients (2, 3). Mismatch repair (MMR) status functions as a major predictor of the efficacy of immune checkpoint inhibitor therapy, which has many limitations: MMR deficiency is found in 10% to 15% of colorectal cancer (CRC) patients, among which only 30% to 50% of them can benefit from the immunotherapy (4–7). Therefore, there is an urgent need to identify new biomarkers that can accurately predict the immunotherapy response of rectal cancer patients, to reveal resistance mechanisms, and to seek potential targets for enhancing immunotherapy efficacy.

The efficacy of immunotherapy for rectal cancer is closely related to the immune infiltration level in RC (2, 8). N6-methyladenosine (m6A) modification, the most common internal modification of messenger RNAs (mRNAs) in eukaryotes (9), is a reversible event modulated by “writers” (*WTAP*, *KIAA1429*, *RBM15*, *RBM15B*, *METTL3*, *METTL16*, and *METTL14*), “erasers” (*FTO* and *ALKBH5*), and “readers” (*HNRNPA2B1*, *HNRNPC*, *YTHDF1*, *YTHDF2*, *YTHDF3*, and *YTHDC1*) (10). Li et al. (11) reported that m6A modification controls T cell homeostasis by targeting the IL-7/STAT5/SOCS pathways. Han et al. (12) reported that YTHDF1-dependent m6A mRNA methylation controls antitumor immunity mediated by dendritic cells (DCs). These findings suggest that m6A modification plays a crucial role in tumor-immune interactions. Meanwhile, several studies indicated altered expression of m6A-related genes in gastrointestinal cancers (13). However, the roles of m6A-related genes in tumor-immune interactions of rectal cancer remain largely unknown.

In this study, we assessed the expression levels of m6A-related genes and their correlations with the prognosis of rectal cancer patients. We observed that *METTL14* expression is positively correlated with overall survival (OS) and tumor immune cell infiltration. Our work suggests an important role of *METTL14* in regulating tumor and immune microenvironment interactions, and identifies *METTL14* as a prognostic biomarker as well as a potential target for enhancing the immunotherapy effect in rectal cancer.

## Materials and Methods

### The Expression Analysis of m6A-Related Genes

The expression levels of m6A-related genes in rectal cancer were obtained from four GEO data sets (GSE123390, GSE87211, GSE60331, GSE68204) and other 3 TCGA data sets from TIMER (14), GEPIA (15) and UCSC Xena (16) for further study (data set A, data set B, and data set C).

### Survival Analysis

The relationship between m6A-related gene expression levels and the prognosis of rectal cancer patients was first explored in the TIMER (14) database (https://cistrome.shinyapps.io/timer/) and GEPIA (15) database (http://gepia.cancer-pku.cn/). TIMER is a web resource that includes 10,897 samples from 32 cancer types from TCGA, and users can explore the clinical effect of genes in the “Survival” module. GEPIA is a web resource that contains 9,736 tumors and 8,587 normal samples from TCGA and the GTEx database, and users can conduct overall survival analysis of an input gene in specific cancers. The threshold for splitting the high-expression and low-expression cohorts can be adjusted. In our study, 50% of patients had a higher expression level than the threshold in both TIMER and GEPIA.

### Immune Infiltration Levels and Immune Marker Set Analysis

The “GENE” module of TIMER allows users to easily explore the association between the expression of a certain gene and the level of infiltration of multiple immune cell types, including macrophages, neutrophils, dendritic cells, B cells, CD4+ T cells, and CD8+ T cells, in a given cancer type. Because tumor purity is an important confounding factor, the first panel of this analysis displayed *METTL14* expression levels against tumor purity. GEPIA provides an interface to conduct gene correlation analysis by using Pearson, Spearman, or Kendall methods; thus, we further explored the correlation between *METTL14* expression and markers of diverse immune cells in the GEPIA database. We chose the Spearman method and used TCGA tumor and TCGA normal data sets. TISIDB (17) (http://cis.hku.hk/TISIDB) is a user-friendly platform to investigate the role of a certain gene in tumor-immune interactions. Tumor-infiltrating lymphocyte (TIL), immunoinhibitor, immunostimulator, chemokine, chemokine receptor, major histocompatibility complex (MHC), immune subtype, and molecular subtype analyses were performed.

### Cell Culture

The rectal cancer cell line cannot be acquired in China amid the pandemic of COVID-19, and human colorectal cancer cell line (HCT116) is generally considered to be a representative colorectal cancer cell line, and we found several studies focusing on rectal cancer, which employed HCT116 to conduct research *in vitro* (Published in *Annals Of Surgery* (18)*, British Journal Of Cancer* (19). We have tried to find normal rectal cell lines when conducting the research as well, and the only normal rectal cell line in ATCC named Hs 680.Rec was not available for international distribution. We found several studies focusing on rectal cancer which employed human normal colorectal epithelial cell line (FHC) to conduct research *in vitro* [Published in *Biomedicine & Pharmacotherapy* (20), *Journal of cellular biochemistry* (21)]. As far as we know, rectal cancer and colon cancer cells have similar biological behaviors (22), so we used HCT116 and FHC in our study. HCT116 and FHC were obtained from the Cell Bank of the Chinese Academy of Sciences and were cultured in RPMI 1640 (GIBCO, Grand Island, NY) supplemented with 10% fetal bovine serum, 100 U/mL penicillin, and 100 µg/mL streptomycin (GIBCO). Cells were maintained in a humidified incubator with a 5% CO_2_ atmosphere at 37°C.

### Arraystar Human m6A-mRNA Epitranscriptomic Microarray Analysis

Total RNA was extracted from HCT116 cells and FHC cells using TRIzol Reagent (Invitrogen, Carlsbad, CA, USA) according to the manufacturer’s instructions and was immunoprecipitated with anti-N6-methyladenosine antibody (Synaptic Systems, 202003). The modified RNAs were eluted from the immunoprecipitated magnetic beads as the “IP.” The unmodified RNAs were recovered from the supernatant as “Sup.” The IP and Sup RNAs were labeled with Cy5 and Cy3, respectively, as cRNAs in separate reactions using the Arraystar Super RNA Labeling Kit (Arraystar, AL-SE-005). The cRNAs were combined and hybridized onto an Arraystar Human mRNA Epitranscriptomic Microarray (8x60K, Arraystar). After washing the slides, the arrays were scanned in two-color channels by an Agilent Scanner G2505C. Agilent Feature Extraction software (version 11.0.1.1) was used to analyze acquired array images. Differential m6A-methylated mRNAs between two samples were identified through fold change filtering and were analyzed for Gene Ontology (GO) and pathway enrichment by using GO (http://www.geneontology.org) and KOBAS (http://kobas.cbi.pku.edu.cn/index.php).

### Retrospective Cohort Patients and Follow-Up

Tissue samples of 150 patients diagnosed with rectal cancer from July 2015 to January 2018 in Xiangya Hospital of Central South University were collected to establish a tissue bank. The demographic characteristics, cancer stages, and pathological reports were obtained from the electronic medical records (EMR) system. A retrospective cohort was performed, as of July 31, 2019, a total of 89 patients were included. Survival analysis, multivariate Cox regression, and the clinical features analysis were conducted after follow-up. This study was reviewed and approved by the Xiangya Hospital Medical Ethics Committee of Central South University.

### Immunohistochemistry Staining

Formalin-fixed, paraffin-embedded tissue array slides were used to detect METTL14 protein expression. Briefly, after deparaffinization and rehydration, citrate buffer (ZLI-9064; ZSGB-BIO, Beijing, China) was used for heat-induced epitope retrieval. Endogenous peroxidase activity was inhibited for 10 min with 3% hydrogen peroxide (reagent 1; PV-9000; ZSGB-BIO). Nonspecific binding was blocked with normal goat serum (ZSGB-BIO) for 15 to 20 min at room temperature. The slides were then incubated overnight at 4°C with METTL14 rabbit polyclonal antibody at a dilution of 1:100 (catalog no. 26158-1-AP; Proteintech, Chicago, USA). Next, the slides were incubated in polymer helper (reagent 2; PV-9000; ZSGB-BIO) for 20 min at 37°C and then incubated with polyperoxidase-anti-mouse/rabbit IgG (reagent 3, PV-9000, ZSGB-BIO) for 20 min at 37°C. 3-3’-diaminobenzidine was used for coloration, and hematoxylin was used for counterstaining.

METTL14 staining was defined as positive when rectal cells showed nuclear staining. A METTL14 staining score was defined by adding the staining intensity score and the positive staining percentage score. The staining intensity was categorized into three grades: score 1, yellow; score 2, light brown; score 3, brown. Positive staining percentage patterns were categorized into four groups: score 1, 0% to 25% staining of rectal cells; score 2, 25% to 50% staining of rectal cells; score 3, 50% to 75% staining of rectal cells; score 4, 75% to 100% staining of rectal cells. The percentage and intensity scores were added, and the results were classified into a high expression group (total scores >4) and a low expression group (total scores ≤4) by using a mean score of 4 as cutoff.

### Agnostic Analysis of *METTL14*

Analysis of differentially expressed genes (DEGs) of *METTL14* expression level (high vs. low) was based on an empirical Bayesian approach using the limma R package (23). An adjusted *P* value less than 0.05 and log2 fold change (log2FC) greater than 1 indicated statistical significance for further gene ontology (GO) enrichment.

The pathway activation levels (PAL) calculated from gene expression data between METTL14-High vs METTL14-Low groups were performed. Both the single sample gene set enrichment analysis (ssGSEA) algorithm (24) and Oncobox library (oncoboxlib) (25) were used for PAL calculation. ssGSEA was performed based on “c2.cp.hallmark.v7.1.symbols” gene sets downloaded from MSigDB database using the “ssGSEA” R package. Oncoboxlib calculates PAL according to Sorokin et al. (25), and it takes a file that contains gene symbols in HGNC format, their expression levels for one or more samples (cases and/or controls) and calculates PAL values for each pathway in each sample (**Table S1**).

### Statistical Analysis

Statistical data were analyzed by using SPSS 20.0 and presented by using GraphPad Prism 7.0. Kaplan-Meier curves and log-rank tests were performed to evaluate the prognostic value of *METTL14* in rectal cancer. Overall survival (OS) was measured from the date of diagnosis to either the date of death or the date of the final follow-up, with final evaluation on July 31, 2019. Multivariate Cox regression analysis was performed to identify whether *METTL14* was an independent prognostic factor of survival in rectal cancer. Multivariate logistics regression analysis was performed to identify the impact factors of *METTL14* expression level (high vs. low) in rectal cancer. The results with a P value less than 0.05 were considered statistically significant if not specified.

## Results

### The Expression Landscape of m6A-Related Genes in Rectal Cancer

**Figure 1:**
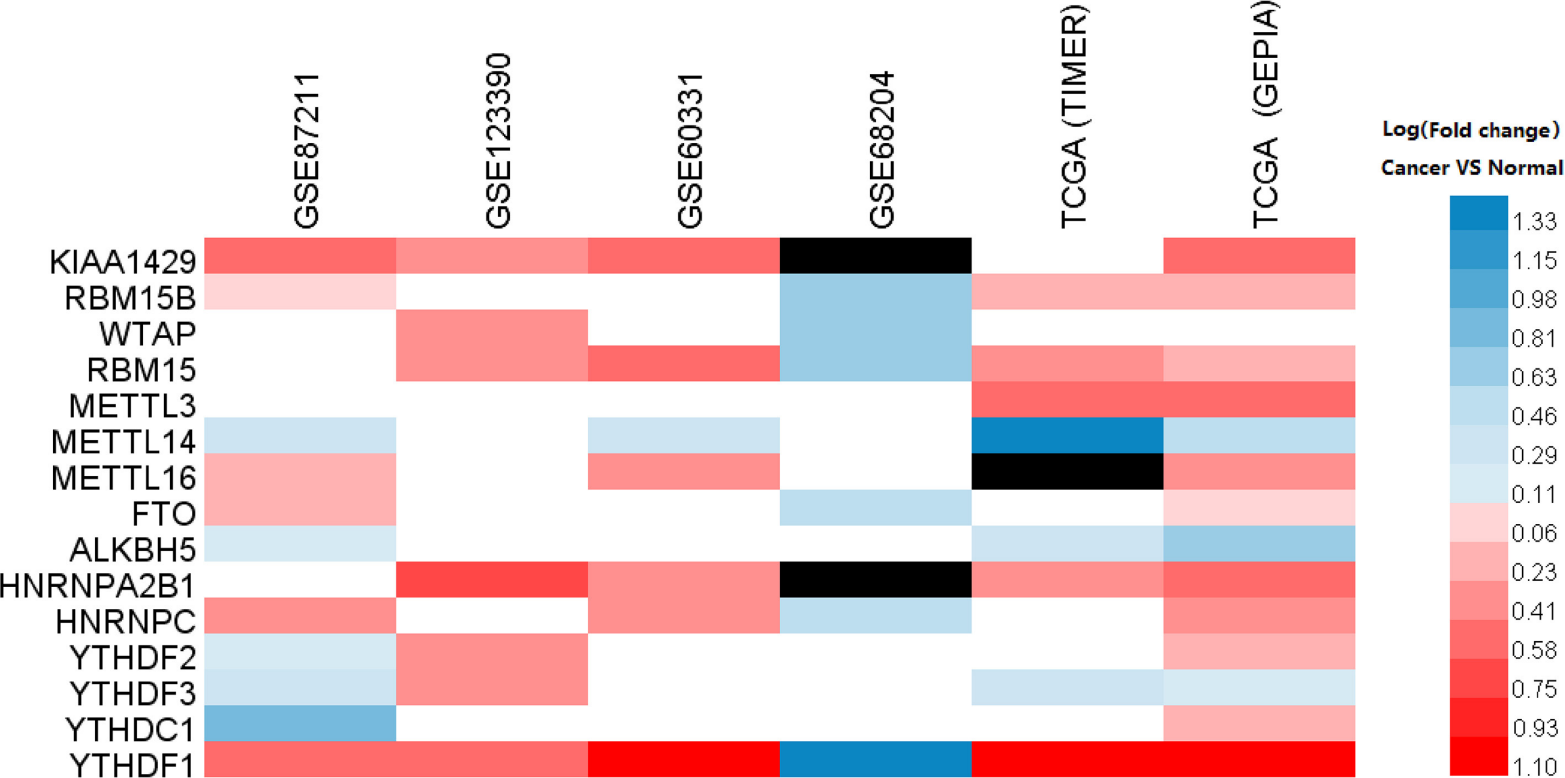
The relative expression of m6A-related genes in rectal cancer. Heatmap showing the alterations in the mRNA expression of m6A-related genes in the TCGA and GEO data sets. The red color indicates upregulated expression; the blue color indicates downregulated expression; the blank indicates no significant changes, and the black color indicates that the related gene is absent in the data sets. The data were statistically analyzed by Student’s *t* test (unpaired, two-tailed).

We used four GEO data sets (GSE123390, GSE87211, GSE60331, GSE68204) and other two TCGA data sets (data sets A and B) to determine the expression level of m6A-related genes in rectal cancer and normal tissues. Besides the data sets without the statistically significant difference, the results showed that compared to normal tissues, the expression levels of *KIAA1429*, *METTL3*, *METTL16*, and *HNRNPA2B1* increased in rectal cancer, and the expression of *METTL14* and *ALKBH5* in rectal cancer was significantly decreased. The expression levels of other nine genes showed different alterations in the tumors using different data sets (**Figures 1** and **S1**).

### The Correlation of the OS Time and the Expression of m6A-Related Genes in Rectal Cancer

**Figure 2:**
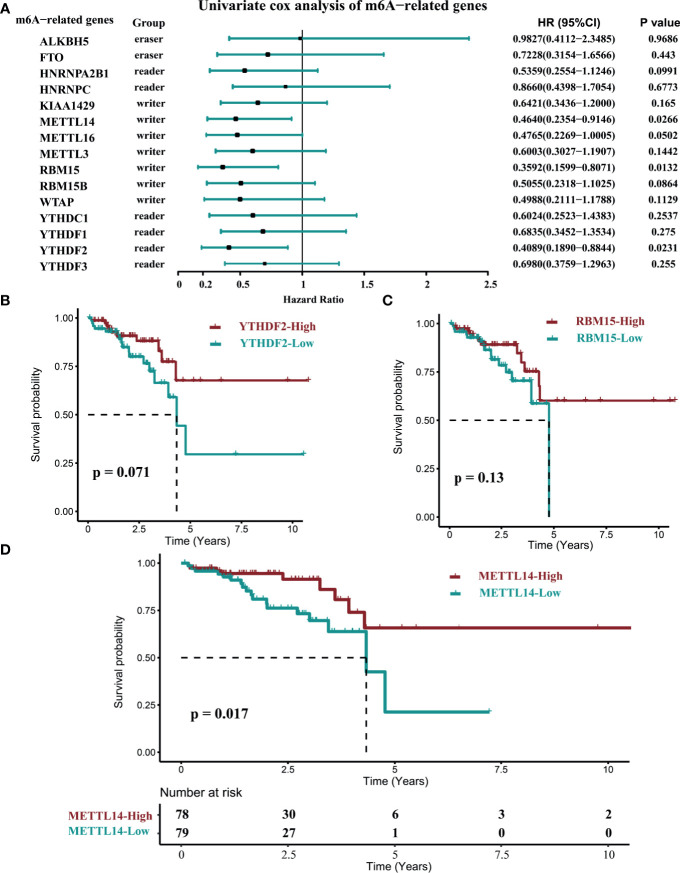
The correlation of the OS time and the expression of M6A-related genes in rectal cancer. (data set C, n=157). **(A)** The univariate COX survival analysis of *METTL14.* **(B)** Log-rank survival analysis of *YTHDF2.* **(C)** Log-rank survival analysis of *RBM15.* **(D)** Log-rank survival analysis of *METTL14.* (cutoff: median expression level).

The mRNA expression levels of m6A-related genes suggested that they might play an important role in rectal cancer. To determine whether the expression level of m6A-related genes is correlated with the prognosis in rectal cancer patients, we selected the latest TCGA data set (data set C), to conduct retrospective studies. In the univariate cox analysis, *RBM15*, *YTHDF2*, and *METTL14* were significantly correlated with prognosis in rectal cancer patients (**Figure 2A**). Nonetheless, in the log-rank test of *RBM15*, *YTHDF2* and *METTL14*, only *METTL14* were correlated with OS in these patients (**Figures 2B–D**). The hazard ratio (HR) for patients with high *METTL14* expression levels was 0.464 (p = 0.0266, **Figure 2A**), which showed that patients with high *METTL14* expression had longer OS time and better prognosis than those with low *METTL14* expression. These results indicated that only *METTL14 *expression was significantly correlated with prognosis in rectal cancer patients, therefore we further observed the impact of *METTL14* expression and m6A modification on the immune cell infiltration in rectal cancer.

### The Correlation of *METTL14* and Immune Infiltrates in Rectal Cancers

**Figure 3:**
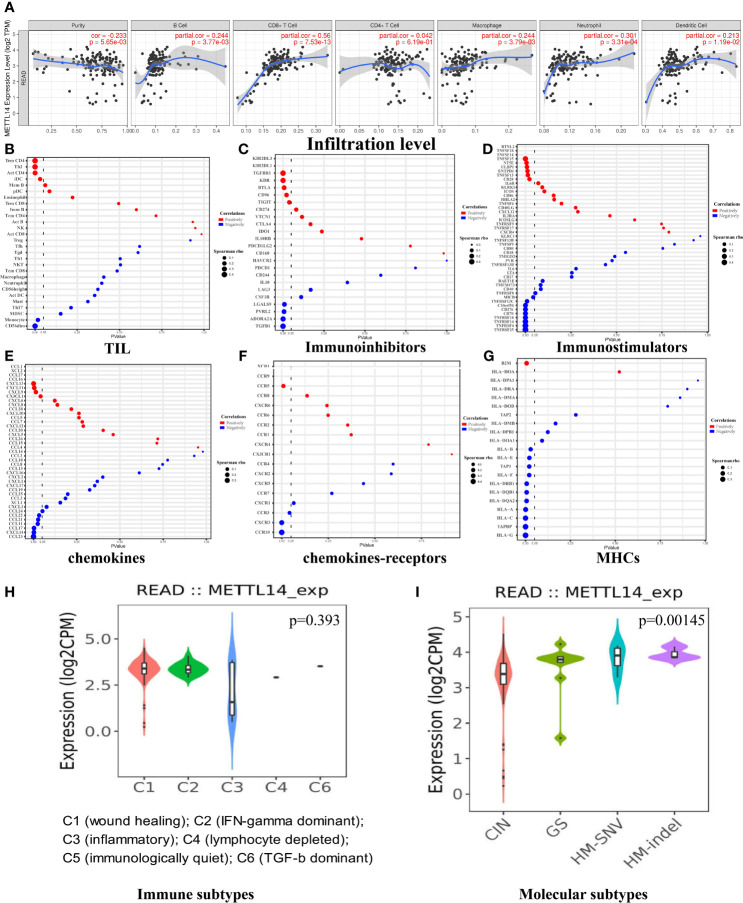
The correlation of *METTL14* and immune infiltrates in rectal cancers. **(A)** The immune infiltrate analysis in the TIMER data set. **(B)** The immune infiltrate analysis in the TISIDB data set. **(C)** The correlation of *METTL14* and immunoinhibitors in the TISIDB data set. **(D)** The correlation of *METTL14* and immunostimulators in the TISIDB data set. **(E)** The correlation of *METTL14* and chemokines in the TISIDB data set. **(F)** The correlation of *METTL14* and chemokine receptors in the TISIDB data set. **(G)** The correlation of *METTL14* and MHCs in the TISIDB data set. **(H)** The relative expression level of *METTL14* in different immune subtypes. **(I)** The relative expression level of *METTL14* in different molecular subtypes. CIN, chromosomal instability; GS, genome stable; HM-SNV, hypermutated single nucleotide variants; HM-indel, hypermutated insertion-deletion.

The tumor immune infiltration level plays a crucial part in affecting the prognosis of rectal cancer patients (26). Therefore, we evaluated whether *METTL14* expression was related to immune infiltration levels in rectal cancer using the TIMER and GEPIA databases. The results from the TIMER database showed that *METTL14* expression level had a significant positive correlation with the levels of B cell (cor. = 0.244, p=0.00377), CD8+ T cell (cor. = 0.56, p<0.0001), macrophage (cor. = 0.244, p=0.00379), neutrophil (cor. = 0.301, p=0.000331), and dendritic cell infiltration (cor. = 0.213, p=0.0119) in rectum adenocarcinoma (READ), while *METTL14* expression was not significantly correlated with CD4+ T cell infiltration (cor. = 0.042, p = 0.619) (**Figure 3A**). To verify the results of the TIMER database analysis, we then investigated the correlation between *METTL14* expression level and immune marker sets of diverse immune cells in the GEPIA database. As expected, the results were basically consistent with those of the TIMER database analysis. Immune markers of CD8+ T cells, B cells, M1 macrophages, M2 macrophages, neutrophils and dendritic cells were positively correlated with *METTL14* expression in READ. In addition, in the GEPIA database, the immune markers of CD4+ T cell, T cell (general), monocyte, tumor-associated macrophage (TAM), T helper 1 (Th1) cell, Th2 cell, Th17 cell, T follicular helper cell (Tfh), regulatory T cell (Treg), natural killer cell, and T cell exhaustion also showed positive correlations with *METTL14* expression levels (**Table 1**).

**Table 1:** The correlation of *METTL14* and the immune infiltrates in rectal cancers.

Description	Gene markers	READ			
		Tumor		Normal	
		Cor.	P	Cor.	P
CD4+ T cell	*CD4*	0.39	9.90E−05	0.52	0.13
CD8+ T cell	*CD8A*	0.38	0.00021	0.38	0.28
	*CD8B*	0.27	0.0099	0.33	0.35
T cell (general)	*CD3D*	0.3	0.0034	−0.042	0.92
	*CD3E*	0.34	0.001	0.21	0.56
	*CD2*	0.35	0.00063	0.13	0.73
B cell	*CD19*	0.3	0.0042	0.21	0.56
	*CD79A*	0.25	0.016	0.35	0.33
Monocyte	*CD86*	0.4	8.3e−05	0.27	0.45
	*CD115 (CSF1R)*	0.43	2.1e−05	0.79	0.0098
TAM	*CCL2*	0.26	0.012	0.81	0.0082
	*CD68*	0.37	3e−04	0.36	0.31
	*IL10*	0.31	0.0024	0.39	0.26
M1 macrophage	*INOS (NOS2)*	0.21	0.044	−0.2	0.58
	*IRF5*	0.1	0.33	−0.079	0.84
	*COX2 (PTGS2)*	0.33	0.0013	0.75	0.018
M2 macrophage	*CD163*	0.31	0.0027	0.75	0.018
	*VSIG4*	0.3	0.0043	0.3	0.41
	*MS4A4A*	0.33	0.0015	0.44	0.2
Neutrophils	*CD66b (CEACAM8)*	−0.042	0.69	−0.25	0.49
	*CD11b (ITGAM)*	0.35	0.00055	0.83	0.0056
	*CCR7*	0.31	0.0023	0.25	0.49
Natural killer cell	*KIR2DL1*	0.094	0.37	−0.18	0.63
	*KIR2DL3*	0.1	0.34	0.29	0.42
	*KIR2DL4*	0.25	0.018	−0.34	0.34
	*KIR3DL1*	0.2	0.053	0.19	0.6
	*KIR3DL2*	0.11	0.3	−0.23	0.53
	*KIR3DL3*	0.072	0.49	0.13	0.73
	*KIR2DS4*	0.039	0.71	0.35	0.32
Dendritic cell	*HLA-DPB1*	0.36	0.00048	−0.091	0.81
	*HLA-DQB1*	0.14	0.17	0.41	0.25
	*HLA-DRA*	0.32	0.0019	−0.091	0.81
	*HLA-DPA1*	0.42	3.1e−05	0.3	0.41
	*BDCA-1 (CD1C)*	0.19	0.073	0.35	0.33
	*BDCA-4 (NRP1)*	0.43	2.1e−05	0.92	0.00047
	*CD11c (ITGAX)*	0.31	0.0024	0.41	0.25
Th1	*T-bet (TBX21)*	0.37	0.00029	0.27	0.45
	*STAT4*	0.37	0.00033	0.63	0.05
	*STAT1*	0.43	1.8e−05	0.78	0.012
	*IFN-γ (IFNG)*	0.28	0.0061	0.17	0.65
	*TNF-α (TNF)*	0.36	0.00036	0.41	0.24
Th2	*GATA3*	0.28	0.0071	0.27	0.44
	*STAT6*	0.3	0.0034	0.65	0.049
	*STAT5A*	0.48	1.1e−06	0.72	0.024
	*IL13*	0.2	0.054	−0.058	0.87
Tfh	*BCL6*	0.33	0.0014	0.71	0.028
	*IL21*	0.31	0.0024	0.0065	0.99
Th17	*STAT3*	0.63	2e−11	0.28	0.43
	*IL17A*	0.12	0.24	−0.12	0.75
Treg	*FOXP3*	0.4	9.4e−05	−0.0061	1
	*CCR8*	0.49	5.9e−07	0.055	0.89
	*STAT5B*	0.6	2.4e−10	0.84	0.0045
	*TGFβ (TGFB1)*	0.26	0.013	0.52	0.13
T cell exhaustion	*PD-1 (PDCD1)*	0.41	5.6e−05	0.26	0.47
	*CTLA4*	0.36	0.00041	0.22	0.54
	*LAG3*	0.28	0.0073	0.39	0.26
	*TIM-3 (HAVCR2)*	0.34	0.001	0.21	0.56
	*GZMB*	0.11	0.32	−0.079	0.84

After confirming that *METTL14 *expression was positively correlated with immune activation signature, we further used the TISIDB database to analyze the correlations between *METTL14* expression and TILs, immunoinhibitors, immunostimulators, chemokines, chemokines-receptors, and major histocompatibility complexes (MHCs). P-values less than 0.05 are considered to be significant. The results of TIL analysis were basically consistent with those of the TIMER and GEPIA database analyses. The levels of CD4+ T cell, Th2 cell, and immature dendritic cell (iDC) infiltration were positively correlated with *METTL14* expression, and the CD4+ T cell infiltration level had the strongest correlation (cor. = 0.4, p < 0.0001). In contrast to the GEPIA results, the level of CD56 dim nature killer cell infiltration (cor.=-0.377, p<0.0001) and monocyte infiltration (cor.= -0.185, p=0.017) was negatively related with *METTL14* expression (**Figure 3B**). The results of immunoinhibitor analysis revealed that *METTL14* expression negatively correlated with four of nine immunoinhibitors’ expression. Among them, transforming growth factor-β1 (*TGF-β1*) had the strongest correlation coefficient (cor. = −0.314, p < 0.0001). Additionally, five out of nine immunoinhibitors were positively correlated, and transforming growth factor-β receptor 1 (*TGF-βR1*) had the strongest correlation (cor. = 0.304, p < 0.0001) (**Figure 3C**). Immunostimulator analysis showed that *METTL14* expression was negatively correlated with most immunostimulators’ expression (9/15), and tumor necrosis factor receptor superfamily 25 (*TNFRSF25*) had the most significant negative correlation (cor. = −0.468, p < 0.0001). However, a few immunostimulators (6/15) had positive correlations, and tumor necrosis factor superfamily-15 (*TNFSF15*) had the strongest correlation (cor. = 0.349, p<0.0001) (**Figure 3D**).

Chemokine analysis showed that all C-C motif chemokine ligand (CCL) family members and *METTL14* expression were negatively correlated. *CCL23* had the strongest correlation (cor. = −0.378, p < 0.0001). Three of four C-X-C motif chemokine ligand (CXCL) family members and *METTL14* expression were positively correlated, and *CXCL13* had the strongest correlation (cor. = 0.271, p=0.000424) (**Figure 3E**). As for the C-C chemokine receptor (CCR) family, chemokine receptor analysis revealed that *CCR10* had a negative correlation with *METTL14* expression (cor. = −0.474, p < 0.0001), and *CCR5 *had a positive correlation with *METTL14* expression (cor. = 0.202, p = 0.00892). In the C-X-C motif chemokine receptor (CXCR) family, there is a negative correlation between *CXCR3* and *METTL14* (cor. = −0.343, p < 0.0001) (**Figure 3F**). MHC analysis showed that all human leucocyte antigen (HLA) family members and *METTL14* expression were negatively correlated, and *HLA-G* had the strongest correlation (cor. = −0.386, p < 0.0001). Antigen peptide transporter (TAP) family members were also negatively correlated with *METTL14* expression, and tapasin binding protein (*TAPBP*) had the strongest correlation (cor. = −0.364, p < 0.0001). However, β-2 microglobulin (*B2M*) was positively correlated with *METTL14* expression (cor. = 0.211, p = 0.00618) (**Figure 3G**).

We also assessed whether *METTL14 *expression was related to immune subtypes (27) and molecular subtypes (28) in READ. The results showed that there was no significant difference in *METTL14 *expression among the six immune subtypes (p = 0.393), while there were differences among molecular subtypes (p = 0.00145). The expression level of *METTL14* in the chromosomal instability (CIN) subtype was the lowest, while *METTL14* expression in genome stable (GS), hypermutated single nucleotide variants (HM-SNV) and hypermutated insertion-deletion (HM-indel) subtypes was higher than that in CIN (**Figures 3H, I**).

### *METTL14* Expression Is a Prognostic Biomarker Correlated With Immune Infiltration in Rectal Cancer

**Figure 4:**
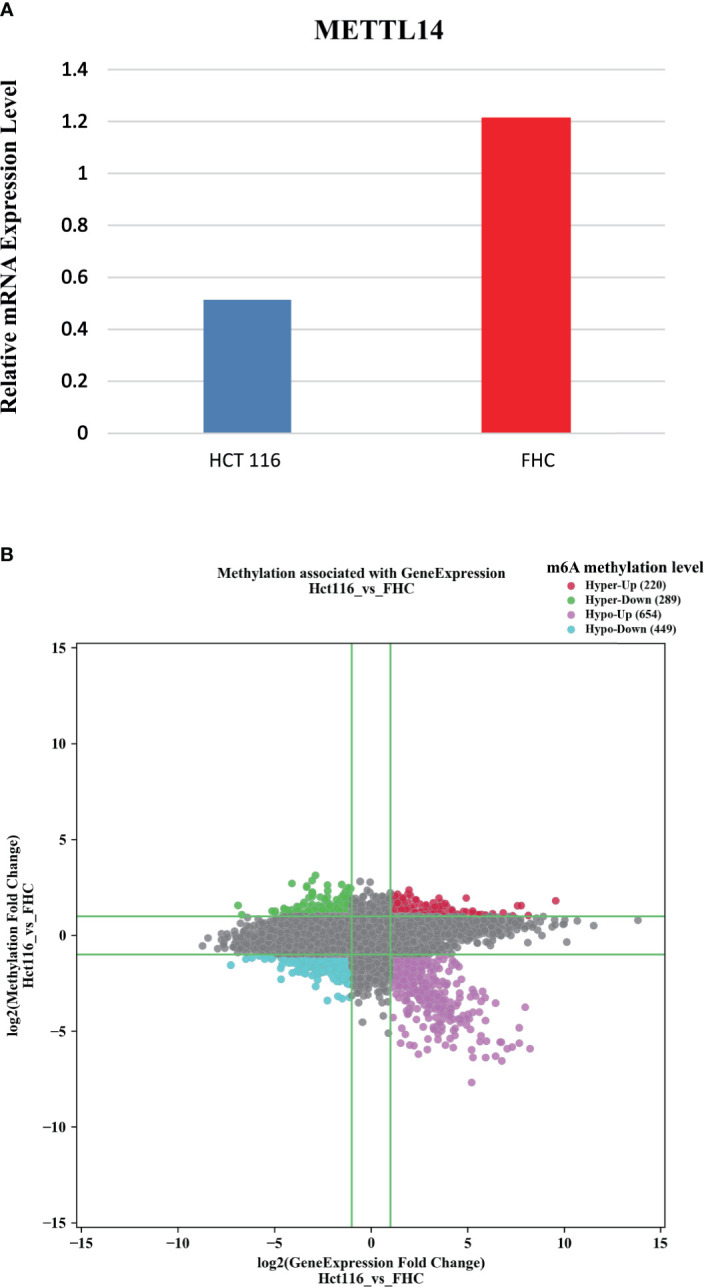
The expression level of *METTL14* and the m6A methylation level in HCT116 and FHC. **(A)** The expression level of *METTL14*. **(B)** The m6A methylation level (Hyper and Hypo: M6A methylation level, Up and Down: Gene expression level).

To verify the impact of *METTL14* on immune infiltration, we cultured HCT116 cells, a human colorectal cancer cell line, and FHC cells, a human normal intestinal epithelial cell line, and conducted Arraystar Human m6A-mRNA Epitranscriptomic microarray analysis. Since *METTL14* functions as an m6A writer gene, its low expression in rectal cancer suggests that the m6A level in rectal cancer cells may be lower than that in normal cells. Our results showed that the expression level of *METTL14* in HCT116 is significantly lower than that in FHC. In HCT116, there are 1103 genes with reduced m6A methylation levels and 509 genes with increased m6A methylation levels. About two thirds of the genes take on reduced m6A methylation levels (**Figure 4**).

**Figure 5:**
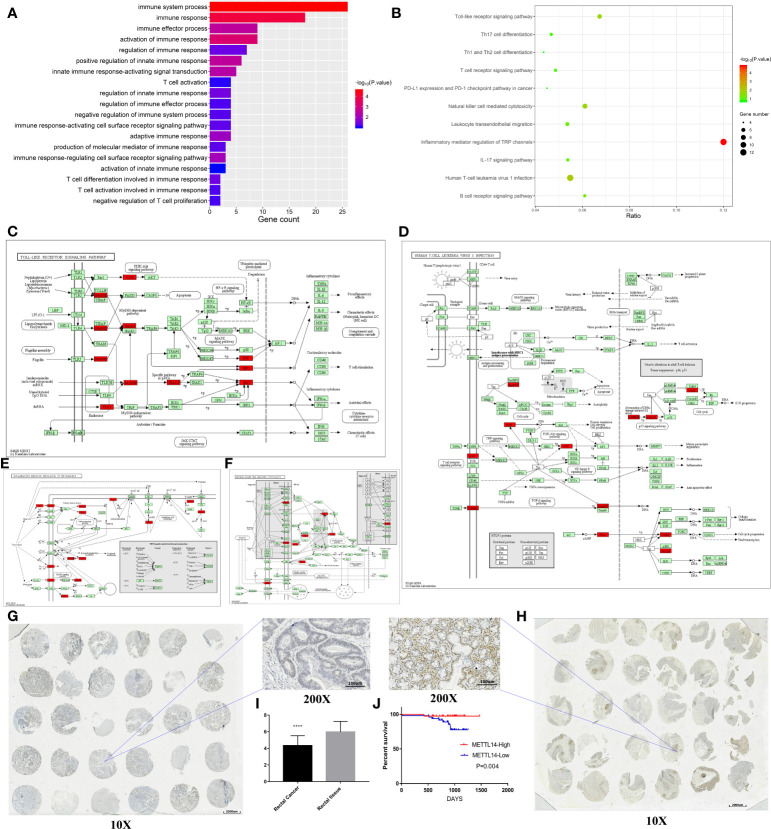
*METTL14* is a prognostic biomarker correlated with immune infiltration in rectal cancer. **(A)** GO analysis of the Arraystar Human m6A-mRNA Epitranscriptomic microarray data on the immunology. **(B–F)** KEGG analysis of the Arraystar Human m6A-mRNA Epitranscriptomic microarray data on the immunology (the enriched genes are highlighted in red in the signaling pathway map). **(G–I)** The expression level of METTL14 in the tissue array. **(J)** Log-rank survival analysis of METTL14 in the retrospective cohort.

We analyzed the microarray results and selected all 1103 genes with reduced m6A methylation levels in HCT116 cells for KEGG and GO analysis. The results of GO analysis showed that these genes were significantly enriched in multiple immune functions, and the five functions with the highest enrichment were immune system process, immune response, immune effector process, activation of immune response, and regulation of immune response (**Figure 5A**). KEGG analysis showed that these genes were enriched in multiple immune-related signaling pathways, and the pathways with the highest enrichment were inflammatory mediator regulation of TRP channels, human T-cell leukemia virus-1 infection, Toll-like receptor signaling pathway, and natural killer cell mediated cytotoxicity. Enriched genes are highlighted in red in the figure (**Figures 5B–F**).

To validate above findings in silico analysis, we conducted a retrospective study. There were 150 rectal cancer tissues and 150 adjacent nontumor tissues in our tissue bank, upon which immunohistochemistry staining for METTL14 was performed using tissue microarrays. We found that 69 patients had high METTL14 expression and 81 patients had low METTL14 expression. Compared with adjacent nontumor tissues, the expression levels of METTL14 protein in rectal cancer tissues significantly decreased (p < 0.0001), which was consistent with the results from the public database (**Figures 5G–I**). A retrospective cohort was performed, and the clinical information of 89 patients was collected. After follow-up, we conducted survival analysis of the cohort. The results showed that patients with high METTL14 expression had longer OS (p = 0.004), and Cox survival analysis identified the expression level of METTL14 as an independent prognostic factor of patients with rectal cancer. The HR value of the patients with high expression levels was 0.077, and the P value was 0.0164. Advanced clinical stages were associated with decreased survival time (HR: 8.392, p=0.0439), and age, gender, and differentiation level were not found to be statistically significant related to survival (**Figure 5J** and **Table 2**). At this time point, the median follow-up time was 30 months, and the median survival time was not yet reached. These results validate METTL14 as a positive prognostic marker in rectal cancer.

**Table 2:** Multivariate Cox regression analysis of OS in Rectal Cancer.

Clinical Feature	Group	HR (95% CI)	P
Age	≤60	1	0.674
	>60	0.773 (0.234–2.563)	
Stage	I–II	1	0.0439
	III–IV	8.392 (1.060–66.439)	
Gender	Male	1	0.0642
	Female	3.098 (0.935–10.260)	
Differentiation level	Low	1	0.765
	High	0.822 (0.228–2.967)	
METTL14	Low	1	0.0164
	High	0.077 (0.010–0.626)	

### Agnostic Analysis of *METTL14*

**Figure 6:**
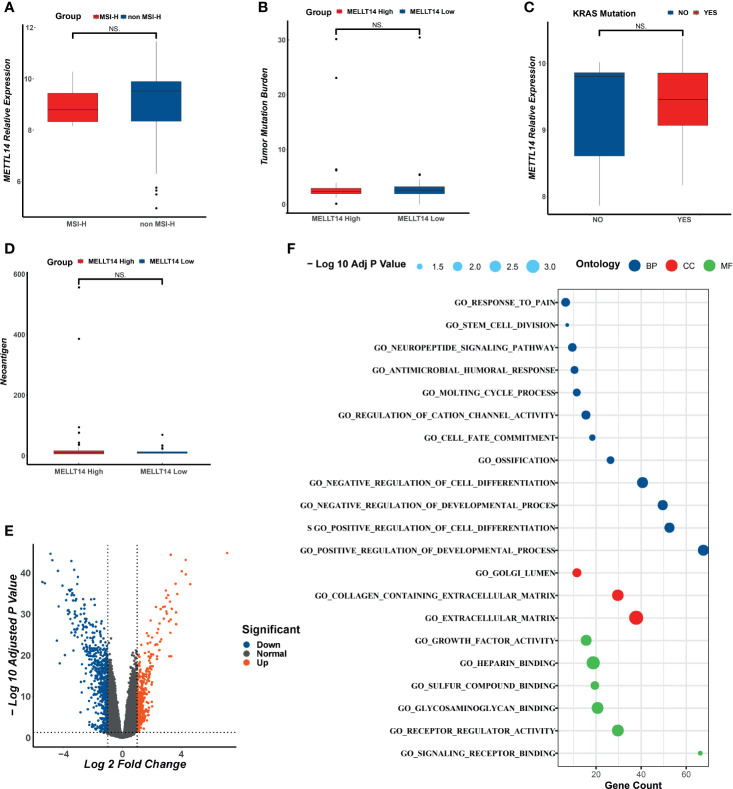
The analysis of clinical feathers and DEGs in different *METTL14* expression group. **(A)** The correlation between MSI status and *METTL14*. **(B)** The correlation between TMB and *METTL14*. **(C)** The correlation between K-RAS mutation status and *METTL14*. **(D)** The correlation between neoantigen and *METTL14*. **(E)** The DEGs of different groups of *METTL14*. **(F)** The GO analysis of DEGs. NS, not significant difference.

To investigate the correlation between clinical features and *METTL14* expression level, in TCGA data set C, we analyzed the correlation between MSI status, TMB, neoantigen, k-Ras mutations and *METTL14* expression level, and there was no statistically significant difference (**Figures 6A–D**). According to the results of IHC, the expression level of *METTL14* were classified into a high expression group and a low expression group. We further conducted a multivariate logistics regression analysis in our validation cohort to quest for the relationship between age, gender, clinical stage, differentiation level, and *METTL14* expression level (high expression group vs. low expression group), and no statistically significant differences were found (**Table 3**).

**Table 3:** Multivariate logistics regression analysis of *METTL14* expression in rectal cancer.

Clinical feature	Group	HR (95% CI)	P
Age	≤60	1	0.743
	>60	0.866 (0.364–2.060)	
Stage	I–II	1	0.733
	III–IV	0.852 (0.337–2.157)	
Gender	Male	1	0.125
	Female	2.053 (0.827–5.266)	
Differentiation level	Low	1	0.291
	High	1.877 (0.601–6.411)	

To further explore the mechanisms of *METTL14*, in TCGA (data set C), we performed DEG analysis for METTL14-High vs METTL14-Low groups and identified a total of 11214 DEGs. There were 1080 DEGs with Log FC >1 and P<0.05. GO analysis showed that the top 20 functions of these DEGs were mostly tied up with tumor-related functions and cell development-related functions (**Figures 6E–F**).

**Figure 7:**
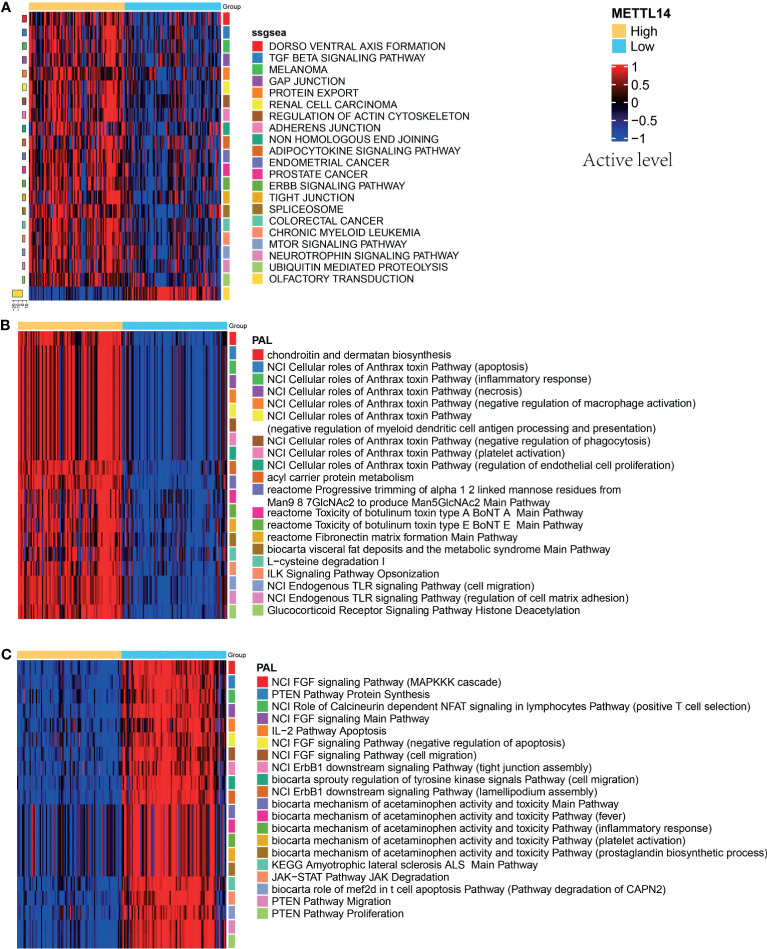
Pathway activation levels analysis in different groups of *METTL14*. **(A)** The top 20 pathways by ssGSEA analysis. **(B)** The top 20 pathways by oncoboxlib analysis in METTL14-High group. **(C)** The top 20 pathways by oncoboxlib analysis in METTL14-Low group.

To further analyze the impact of the expression level of *METTL14* on signal pathways, we used ssGSEA algorithm (24) and Oncobox library (oncoboxlib) (25) to compare PAL calculated from gene expression data between METTL14-High and METTL14-Low groups. The ssGSEA results showed that the top 20 activated pathways were basically tumor-related pathways, including immunity pathways (TGF-β), cell junctions pathway (adherens junction), and metabolism pathway (mTOR signaling pathway) (**Figure 7A**). The results in oncoboxlib included more than 3,000 signal pathways, which was different from ssGSEA. In METTL14*-*High group, the top 20 pathways with high PAL were primarily immune-related, migration-related and apoptosis-related pathways (**Figure 7B**). In METTL14*-*Low group, the top 20 pathways with high PAL were mainly related to the negative regulation of apoptosis, cell migration, and immunity (**Figure 7C**). Compared with ssGSEA algorithm, the results from oncoboxlib reflect the biological function of *METTL14* more properly, which is consistent with the results of our clinical survival cohort. In sum, *METTL14* is a good prognostic factor in rectal cancer.

## Discussion

Notably, decreased *METTL14* expression was found to be significantly correlated with poor prognosis in rectal cancer patients. Furthermore, our study indicates that the low expression of the m6A writer gene *METTL14* in rectal cancer may lead to a decrease in m6A RNA modification, thus reducing the level of immune cell infiltration and resulting in poor prognosis. This mechanism for our findings needs further validation.

The analysis of the expression of m6A-related genes in rectal cancer showed that the expression levels of *KIAA1429*, *METTL3*, *METTL16*, and *HNRNPA2B1* were higher and the expression levels of *METTL14* and *ALKBH5* in rectal cancer were significantly lower than those in normal tissues, showing that aberrant expression of m6A regulatory genes commonly occurs in rectal cancer.

Although many m6A-related genes were dysregulated in rectal cancer, only *METTL14* was related to the prognosis of rectal cancer patients. The TCGA data sets and our retrospective cohort confirmed that *METTL14* had a lower expression level in rectal cancer tissues than in normal adjacent tissues, and that patients with low *METTL14* expression had shorter OS times than those with high *METTL14* expression, which is consistent with previous study (29). Multivariate survival analysis (COX) of our cohort revealed *METTL14* as an independent prognostic factor in rectal cancer patients. These findings strongly suggest that *METTL14* is a prognostic biomarker in rectal cancer.

Immunogenomic analysis of over 10,000 tumors in TCGA data sets revealed that tumor immune landscapes differ greatly between and within cancer types (30, 31). CRC development is driven by Wnt/Myc hyperactivation, KRAS/BRAF mutation, genetic instability and accompanied by progressive immunosuppressive tumor microenvironment (TME) (32). CRCs have poor response to immune checkpoint inhibitors, expect for those with MMR deficiency or high levels of T-cell infiltration (32). Han D. et al. (12) reported that m6A-related genes can affect tumor progression through the tumor immune microenvironment, but their roles in rectal cancer remain unknown. Our data support a potential role of *METTL14* in rectal cancer with increased antitumor immunity. Using the TIMER, GEPIA, and TISIDB databases, our results revealed the positive correlation of the expression level of METTL14 with the immune infiltration level in rectal cancer, including gene signature of CD8+ T cell, CD4+ T cell, B cell, DCs, macrophages, neutrophils and Th2, which were consistent in at least two databases mentioned above. It is well established that increased immune infiltration is conducive to more favorable prognosis in many types of cancers such as melanoma, head and neck, breast, bladder, urothelial, ovarian, renal, prostatic, lung, chordoma, and colorectal cancer (26, 33–35), and infiltrating immune cells mainly consist of the T lymphocyte family, such as CD8+ T cell and CD4+ T cell (36–39). In addition to T cells, B cells also play an important role in antitumor immunity. Some studies have shown that B cells, the key cells in the humoral immune response, can act on cells and molecules in the tumor microenvironment by producing antibodies, thus suppressing tumor progression. However, B cells can also inhibit antitumor immune responses by secreting some cytokines, resulting in poor prognosis (40). Therefore, B cells have a complex part in tumor immunity. DCs can process tumor antigens and present them to T cells, thus exerting antitumor effects. Some cancer vaccines are based on DCs (41, 42). Vyrynen JP et al. (43) found macrophage polarization rather than absolute overall density was associated with colorectal cancer mortality, with M1-like and M2-like macrophages showing opposite effects. Lu Y et. al (44) found CD16 expression on neutrophils in peripheral blood was a good prognostic marker for predicting efficacy of capecitabine in CRC patients. Increased Th2 populations correlated with longer survival in female patients with CRC (45). In conclusion, *METTL14* may affect the clinical outcome of rectal cancer patients by regulating the immune infiltration level in the microenvironment.

We expanded our in silico analysis to examine the correlation of *METLL14* expression with several classes of immune modulators. *METTL14* expression showed a negative association with *TGFβ1*, which is known to promote tumor immune tolerance by upregulating PD1 expression on T cells (46). The expression level of *TGF-βR1*, the receptor of TGF-β1, was positively correlated with *METTL14* expression, which might be explained by a compensatory increase caused by the decrease in *TGF-β1* expression levels. Immunostimulators analysis revealed that *METTL14* expression was positively correlated with the expression level of some immunostimulators, and *TNFSF15* had the strongest correlation coefficient. Zhao et al. (47) found that *TNFSF15* had great importance in the DC-involved Th9 differentiation process, and Th9 cells contributed to antitumor immunity. However, as an immunostimulator, *TNFRSF25* had a negative correlation with *METTL14* expression. TNFRSF25, also known as death receptor 3, is mainly expressed on the surface of T cells. Interestingly, *TNFRSF25* plays a complex dual role in tumors: on the one hand, it binds to its ligand TL1A to activate T cells and inhibit tumor progression (48); on the other hand, through the PI3K/NF-kB pathway, it can reduce the apoptosis of colon cancer cells and promote tumor proliferation and metastasis (49).

Chemokine analysis showed that CCL family members, especially *CCL23*, showed strong negative correlation with *METTL14* expression, whereas most CXCL family members, especially *CXCL13*, showed strong positive correlation. Hannah H. Yan et al. (50) found that *CCL23* was mainly secreted by CD33+ myeloid cells and could act on the TGF-β signaling pathway together with CCL9 to promote the progression and metastasis of breast cancer. The role of *CXCL13* in tumors is controversial. It can not only promote the invasion and metastasis of tumor cells, but also activate immune cells, increase the immune infiltration level in cancer, and inhibit tumor proliferation (51, 52). Chemokine receptor analysis showed that *CCR5 *had a weak positive correlation with *METTL14* expression, and *CCR10* had a negative correlation with *METTL14* expression. *CCR5* is widely involved in tumor proliferation and metastasis, and anti-CCR5 therapy has made some progress in various tumors (53), suggesting that *CCR5* and *METTL14* play the opposite role in cancer. However, due to the weak correlation (cor. = 0.202), their relationship needs further verification. Hao-yu Lin et al. (54) found that *CCR10* promoted the metastasis and invasion of breast cancer through the ERK1/2/MMP-7 signaling pathway. In the CXCR family, *CXCR3* had a negative correlation with *METTL14*. The role of *CXCR3* in tumors is also under dispute. Some studies pointed out that *CXCR3* promoted tumor proliferation and invasion through autocrine mechanisms, while other studies showed that *CXCR3* inhibited tumor growth by promoting the differentiation of immune cells and activating immune cells (55).

The results of MHC analysis showed that all HLA family members and *METTL14* were negatively correlated in terms of expression, and *HLA-G* had the highest correlation coefficient. *HLA-G* has been reported to be a key molecule in tumor immune tolerance and is associated with poor prognosis in cancer patients (56). *TAPBP*, a member of the TAP family, has the strongest negative correlation with *METTL14*. Inconsistent with our results, previous studies have shown that *TAPBP* participates in the antigen presentation pathway, mediates the immune process, and inhibits tumor proliferation (57), suggesting complex interaction mechanism between *METTL14* and *TAPBP*. *B2M* was the only MHC gene that was significantly positively correlated with *METTL14*. Several studies have reported that the loss or mutation of the *B2M* gene was one reason for tumor immune escape. Considering that B2M loss is a mechanism for resistance to anti-PD-1 therapy, *METTL14* may be associated with anti-PD-1 efficacy (58).

The expression level of *METTL14* in rectal cancer varies according to molecular subtype. The chromosomal instability (CIN) subtype had a low expression level of *METTL14*, which was consistent with previous studies. CIN can cause tumor immune escape, drug resistance, and metastasis, leading to poor clinical outcomes (59).

In conclusion, the impact of *METTL14* on the immune infiltration level of rectal cancer may be related to *TGFβ1*, *TNFSF15*, *CCL23*, *CCR10*, *HLA-G*, *B2M*, and *CIN*. These findings suggest an interesting possibility that *METTL14* expression is associated with anti-PD-1 efficacy. Further studies are needed to determine the cause of reduced *METTL14* expression, and how it contributes to suppressive immune TME through abovementioned mediators.

As an m6A writer gene (60), *METTL14* may affect the survival of rectal cancer patients by downregulating m6A methylation levels. Human cell m6A-mRNA epitranscriptomic microarray analysis showed that m6A methylation levels of over 1,000 genes were reduced in rectal cancer cells. Further analysis showed that GO terms and pathways were significantly enriched in immune-related functions and pathways. In the human T-cell leukemia virus-1 infection signaling network, the TGF-β pathway, NF-kB pathway, TNF pathway, P53 pathway, and PI3K/AKT pathway were enriched, which was consistent with the results we mentioned earlier. Macrophages and mast cells are the main cells involved in the inflammatory mediator regulation of TRP channels, and T cells are the major activated cells in the Toll-like receptor signaling pathway, which is consistent with the infiltrating immune cells mentioned before. However, in the natural killer cell-mediated cytotoxicity signal network, we found that *METTL14* was negatively correlated with the CD56 dim nature killer cell infiltration level; therefore, a negative regulatory relationship may exist between *METTL14* and NK cells. Since the m6A microarray results are based on the single experiment, so the further validation is needed. Our research preliminarily explores the mechanism by which *METTL14* regulates the immune infiltration of rectal cancer and provides inspiration for further research.

The mechanisms of *METTL14*’s role in rectal cancer are not only associated with immune system. The agnostic analysis between METTL14-High and METTL14-Low groups showed that besides immunity, the expression of *METTL14* was also widely associated with cell apoptosis, adhesion, migration, and cell development process, suggesting that *METTL14* plays a multifaceted role in rectal cancer, which needs further validation. Some studies have reported that *METTL14* inhibited tumorigenicity, CRC cells growth, invasion, migration, and metastasis, which is consistent with our findings (61–63).

## Conclusion

*METTL14* expression level is an independent prognostic factor in rectal cancer and is positively correlated with the immune infiltration level. Furthermore, we identified *METTL14* as a potential target for enhancing immunotherapy efficacy in rectal cancer.

## Data Availability Statement

The original contributions presented in the study are included in the article/**Supplementary Material**. Further inquiries can be directed to the corresponding authors.

## Ethics Statement

The studies involving human participants were reviewed and approved by Xiangya Hospital Medical Ethics Committee of Central South University. The patients/participants provided their written informed consent to participate in this study. Written informed consent was obtained from the individual(s) for the publication of any potentially identifiable images or data included in this article.

## Author Contributions

SZ, HS, and JY designed the study. SZ and HS supervised the study. CC and JL performed the experiments. CC, JL, QH, YP, SL, KL, CG, XW, YC, and YH analyzed and interpreted the data. ES and CC performed the statistical analysis. CC, JL, SZ, HS wrote the manuscript. All authors contributed to the article and approved the submitted version.

## Funding

This study was supported by grants from the National Key R & D Program of China (No. 2018YFC1313300) and the National Natural Science Foundation of China (81070362, 81172470, 81372629, 81772627, 81874073, and 81974384), two key projects from the Nature Science Foundation of Hunan Province (2015JC3021 and 2016JC2037), the Fundamental Research Funds for the Central Universities of Central South University (2020zzts273) and a China Cancer Elite Team Innovative Grant (201606).

## Conflict of Interest

The authors declare that the research was conducted in the absence of any commercial or financial relationships that could be construed as a potential conflict of interest.

## Supplementary Material

Supplementary Figure 1 The relative expression of m6A-related genes in rectal cancer. **(A)** The expression level of *METTL14* in the TIMER data set (n = 166). **(B)** The expression level of *METTL14* in the GEPIA data set (n = 92). The data were statistically analyzed by Student’s t test (unpaired, two-tailed).

## Abbreviations

RC, rectal cancer; m6A, N6-methyladenosine; CNV, copy number variation; MMR, mismatch repair; CRC, colorectal cancer; DC, dendritic cells; OS, overall survival; READ, rectum adenocarcinoma; TIL, tumor infiltrating lymphocyte; Th, T helper; Tfh, T follicular helper cell; Treg, regulatory T cell; MHC, major histocompatibility complexes; iDC, immature dendritic cell; *TGF-β1*, transforming growth factor-β1; *TGF-βR1*: transforming growth factor-β receptor 1; *TNFSF15*: tumor necrosis factor superfamily-15; *TNFRSF25*, tumor necrosis factor receptor superfamily 25; CCL, C-C motif chemokine ligand; CXCL, C-X-C motif chemokine ligand; CCR, C-C chemokine receptor; CXCR, C-X-C motif chemokine receptor; *HLA*, human leucocyte antigen; *TAP*, antigen peptide transporter; *TAPBP*, tapasin binding protein; *B2M*, β-2 microglobulin; CIN, chromosomal instability; GS, genome stable; HM-SNV, hypermutated single nucleotide variants; HM-indel, hypermutated insertion-deletion.
