# Supplementary figures and images for: M6A “Writer” Gene METTL14: A Favorable Prognostic Biomarker and Correlated With Immune Infiltrates in Rectal Cancer
Source: Front Oncol. 2021 Jun 17;11:615296. doi: 10.3389/fonc.2021.615296 (PMC8247640; doi:10.3389/fonc.2021.615296)

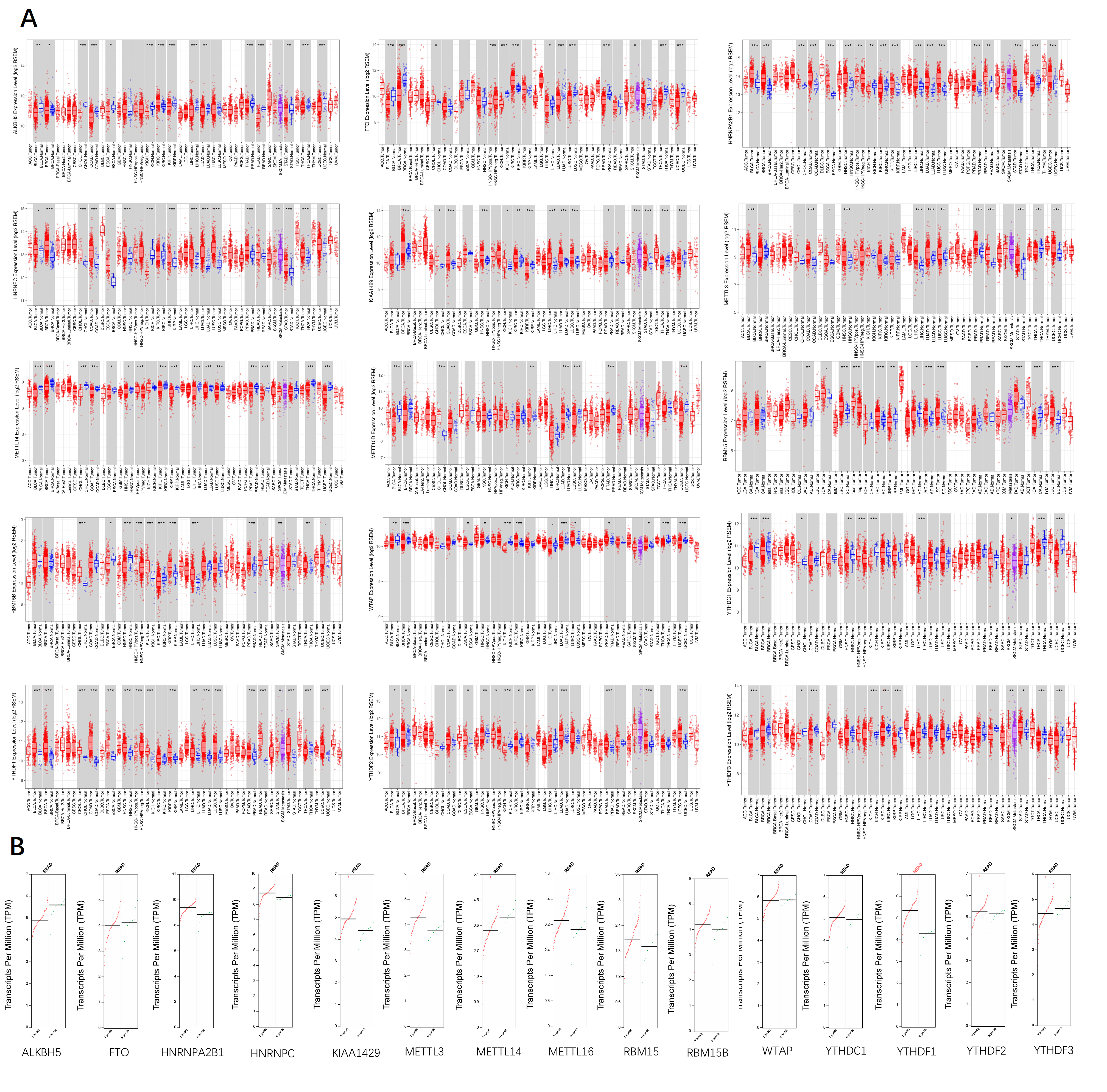

Supplement: Supplementary Figure 1 — The relative expression of m6A-related genes in rectal cancer. (A) The expression level of METTL14 in the TIMER data set (n = 166). (B) The expression level of METTL14 in the GEPIA data set (n = 92). The data were statistically analyzed by Student’s t test (unpaired, two-tailed). [file Image_1.tif]
